# Systematic review and meta-analysis of machine learning-based prediction models for readmission risk after total hip and knee arthroplasty

**DOI:** 10.3389/fmed.2026.1855638

**Published:** 2026-07-15

**Authors:** Jiabin Feng, Min Ma, Changliang Ou, Kaiwei Zhang

**Affiliations:** 1 College of Traumatology and Orthopedics, Guizhou University of Traditional Chinese Medicine, Guiyang, Guizhou, China; 2 Department of Endocrinology, The Second People’s Hospital of Guizhou Province, Guiyang, Guizhou, China

**Keywords:** artificial intelligence, machine learning, Patient Readmission, risk prediction, systematic review, total hip arthroplasty, total knee arthroplasty

## Abstract

**Background:**

Postoperative readmission is a critical metric after total hip and knee arthroplasty (THA/TKA). While machine learning (ML) models for predicting readmission risk are proliferating, the stability of their performance and the robustness of their methodology remain contentious. This study aimed to systematically review and quantitatively synthesize the evidence base for ML-based readmission prediction after THA/TKA.

**Methods:**

A systematic search was conducted across PubMed, Embase, Cochrane Library, and Web of Science from inception to December 31, 2025. Studies developing or validating ML models for THA/TKA readmission were included. Model performance (C-statistic/AUC) was extracted. Study quality was assessed using the PROBAST + AI tool. A multivariate random-effects meta-analysis was performed to pool C-statistics and quantify heterogeneity, with subgroup analyses stratified by study design, surgery type, prediction timeframe, and algorithmic class.

**Results:**

Fifteen studies (57 distinct models) were included. The pooled C-statistic was 0.76 (95% CI: 0.71–0.81). However, extreme heterogeneity (*I*^2^ = 99.9%) rendered this point estimate of limited clinical utility; the 95% prediction interval (0.38–0.94) highlighted profound outcome unpredictability. Subgroup analyses revealed significant moderators: single-center models showed optimistically higher performance (0.86) compared to multicenter models (0.65), and THA-specific models yielded higher estimates than TKA-specific models, although these findings were derived from few studies and should be interpreted as exploratory. Advanced ML algorithms did not demonstrate consistent superiority over traditional logistic regression. Crucially, the PROBAST + AI assessment identified a high risk of bias in the majority of studies, primarily due to analytical shortcomings and a universal lack of model recalibration.

**Conclusion:**

The current body of evidence for ML-based readmission prediction after THA/TKA is characterized by extreme heterogeneity and high methodological bias, severely constraining clinical utility. The inability to pool calibration metrics represents a critical evidence gap. Future research must prioritize multi-institutional validation, stringent adherence to reporting standards (e.g., TRIPOD-AI), and the mandatory transparent reporting of both discrimination and calibration metrics to realize any potential clinical benefit.

**Systematic review registration:**

https://www.crd.york.ac.uk/PROSPERO/view/1305608, identifier CRD420261305608.

## 1 Introduction

When conservative treatments fail to alleviate severe joint dysfunction caused by conditions such as osteoarthritis or rheumatoid arthritis, joint arthroplasty represents a definitive and crucial therapeutic solution. Among these procedures, total hip arthroplasty (THA) and total knee arthroplasty (TKA) are the most widely performed and representative interventions (1). These surgeries involve implanting customized prostheses, typically fabricated from materials such as metals, high-molecular-weight polyethylene, and ceramics, to replace the diseased joint, with the primary goals of relieving pain, correcting deformity, and restoring function. They play an indispensable role in improving patients’ quality of life (2).

In recent years, machine learning (ML) has gained significant prominence in medical prediction (3, 4). By analyzing structured data, including patient demographics, biomarkers, medical history, and anesthetic records, and employing algorithms to explore relationships between features and outcomes, ML models can identify features most strongly associated with the target event, thereby enhancing prediction accuracy. Postoperative readmission following THA/TKA, a key metric for evaluating surgical efficacy and patient prognosis, has consequently attracted increasing research attention (5–19). Although numerous studies have applied machine learning (ML) for THA/TKA readmission prediction, substantial heterogeneity persists in reported performance and predictors. Existing models predominantly incorporate demographic, comorbidity, and procedural variables, yet largely overlook neurocognitive factors that are increasingly recognized as key drivers of postoperative complications and unplanned readmission. While broad reviews of AI in orthopedics exist (3, 11, 20), they lack the granularity required for arthroplasty-specific risk stratification and fail to address the methodological inconsistencies driving performance variability. Critically, no prior synthesis has quantified the extreme between-study heterogeneity or systematically compared the generalizability of single-center models against multicenter cohorts. Furthermore, the putative advantage of complex algorithms over traditional logistic regression remains unverified in this domain. Therefore, this systematic review and meta-analysis aims to bridge this gap by providing a granular evaluation of ML model performance, with a specific focus on quantifying heterogeneity, comparing algorithmic efficacy, and identifying consistent predictors of readmission. Our findings seek to guide the development of robust, clinically applicable models that avoid common pitfalls such as optimism bias and calibration errors induced by improper class imbalance handling. Therefore, this study aims to conduct a systematic review and meta-analysis of the existing literature on ML-based prediction models for readmission after THA/TKA, to summarize the current evidence and provide a reference for future model development and clinical application.

## 2 Materials and methods

This systematic review and meta-analysis was conducted and reported in accordance with the Preferred Reporting Items for Systematic Reviews and Meta-Analyses (PRISMA) statement (21). Its protocol was prospectively registered with the International Prospective Register of Systematic Reviews (PROSPERO; registration number: CRD420261305608). The study design adhered to the PICOTS framework. The target population comprised patients undergoing total hip or knee arthroplasty (THA/TKA). The predictive intervention of interest was the application of a machine learning (ML)-based model to estimate the risk of postoperative readmission. The primary outcome of interest was the discriminative accuracy of these models for predicting readmission, quantified by the area under the receiver operating characteristic curve (AUC) (22), measured from discharge to a specified postoperative interval. As the focus was on evaluating the models themselves, no direct comparator was required. The intended application setting was the hospital environment.

### 2.1 Literature search

A comprehensive literature search was conducted in PubMed, the Cochrane Library, Embase, and Web of Science from their inception to December 31, 2025. The search strategy combined Medical Subject Headings (MeSH) and free-text terms covering three key concepts: arthroplasty (“Total Joint Arthroplasty,” “Total Knee Arthroplasty,” “Total Hip Arthroplasty,” “Joint Replacement”), readmission (“Readmission,” “Rehospitalization,” “Re-admission”), and artificial intelligence-based predictive modeling (“Machine Learning,” “Deep Learning,” “Artificial Intelligence,” “Predictive Model,” “Risk Prediction,” “Prediction Model,” “Risk Model,” “Algorithm”). The detailed search strings for each database are provided in Supplementary material.

### 2.2 Inclusion and exclusion criteria

Studies were included according to the following criteria: (1) The study population consisted of patients who underwent THA or TKA. (2) The primary or secondary outcome included postoperative readmission. (3) The study developed or validated a multivariable prediction model for readmission. For the purposes of this review, “machine learning (ML)-based models” were operationally defined broadly to include the full spectrum of supervised learning algorithms. This encompassed both traditional statistical approaches (e.g., Logistic Regression, LASSO) and advanced ML algorithms (e.g., Random Forest, Support Vector Machines, Artificial Neural Networks, Gradient Boosting Machines). Studies were excluded only if they failed to construct a multivariable prediction model (e.g., only reported univariate associations or descriptive statistics). (4) The publication was an original research study in a peer-reviewed journal. (5) The full text was available in English.

Studies were excluded if they were: (1) conference abstracts, reviews, commentaries, case reports, or dissertations; (2) not accessible in full text or were duplicate publications; (3) studies that failed to develop or validate a multivariable prediction model, such as those involving only univariate risk factor analysis or basic statistical comparisons; (4) studies reporting composite endpoints from which data specific to readmission could not be separately extracted; or (5) studies that did not report key model performance metrics (such as AUC) or the follow-up duration.

### 2.3 Study selection and data extraction

Two reviewers independently screened the titles, abstracts, and full texts of retrieved records against the pre-defined eligibility criteria. Disagreements were resolved through discussion or, if necessary, by consulting a third reviewer. For each included study, data were extracted independently by two reviewers using a standardized form based on the CHARMS (Checklist for critical Appraisal and data extraction for systematic reviews of prediction modeling studies) checklist (5), which provides a structured framework for extraction. The extracted information included: first author, publication year, study population, predictors, method for handling class imbalance, type of surgery (THA, TKA, or both), postoperative observation period (days), total sample size, number of readmission events, models developed, and the reported performance metrics (e.g., AUC).

### 2.4 Quality assessment (risk of bias)

The risk of bias and concerns regarding the applicability of the included prediction modeling studies were independently assessed by two reviewers using the PROBAST + AI tool (Prediction model Risk Of Bias ASsessment Tool for studies developing, validating, or updating a multivariable prediction model, with an extension for Artificial Intelligence) (23). This tool, an update and extension of the original PROBAST, evaluates four domains (participants, predictors, outcome, and analysis) for both model development and validation. Prior to formal assessment, the two reviewers, both trained in evidence-based medicine, conducted a calibration exercise on the PROBAST + AI tool items. They then performed a pilot assessment on four randomly selected studies to ensure a consistent understanding and application of the criteria. The inter-rater reliability, measured by Cohen’s kappa, ranged from 0.464 to 0.868 for the risk-of-bias domains and was 1.00 for all applicability domains (see Supplementary Table 1) (24). Any discrepancies in the final assessments were resolved through team consensus.

### 2.5 Statistical analysis

A meta-analysis of the C-statistic requires an estimate of its variance. Among the 15 included studies, 7 provided confidence intervals. For the remaining 8 studies, 95% confidence intervals were calculated using the standard error of the C-statistic and assuming a normal approximation. The meta-analysis was performed using the R package “metamisc,” designed for the meta-analysis of prediction model performance measures (25). Crucially, to account for the non-independence of multiple models reported within the same study, we employed a multivariate random-effects model (also known as a multilevel model). This model structure explicitly partitions the residual variance into between-study and within-study components, adjusting the weights accordingly to prevent the artificial inflation of precision. A random-effects model was used to pool the C-statistics, with weighting based on the within-study error variance. The 95% confidence interval for the summary C-statistic was estimated using the Hartung-Knapp-Sidik-Jonkman method (26). To verify the robustness of the findings, subgroup analyses were planned based on surgery type (THA only, TKA only, or combined), data source (single-center vs. multicenter), method for handling class imbalance, length of postoperative follow-up (days), and model category. Potential small-study bias was assessed via a funnel plot, visually inspecting the scatter of the reported C-statistic against its standard error for asymmetry. The statistical significance of the asymmetry was tested using the weighted Egger’s regression test (27).

## 3 Results

### 3.1 Literature search and selection

**FIGURE 1:**
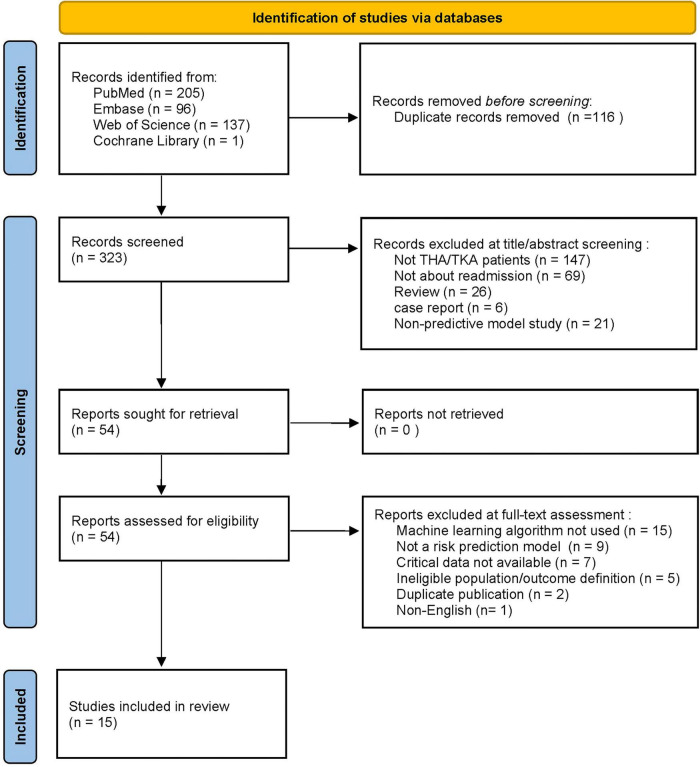
PRISMA flow diagram.

The initial database search yielded 439 records. Following the removal of duplicates and the screening of titles and abstracts, the full texts of 54 potentially eligible studies were retrieved and assessed. Ultimately, 15 studies satisfied all inclusion criteria and were incorporated into this systematic review and meta-analysis. The detailed study selection process is illustrated in the PRISMA flow diagram (Figure 1).

### 3.2 Characteristics of included studies

The 15 included studies all focused on developing machine learning (ML) models to predict readmission risk following total hip arthroplasty (THA) or total knee arthroplasty (TKA). Key characteristics are summarized in Tables 1, 2 (5–19).

**TABLE 1:** Study characteristics.

Study	Study population	Predictors	Class imbalance handling
Mohammadi et al. (5)	Hip/knee arthroplasty EHR patients, Partners Healthcare, 2006–2016.	Comprehensive clinical and demographic variables (e.g., demographics, medical history, vital signs, laboratory results, comorbidities, medications, procedures, diagnoses, admission details).	Undersampling
Slezak et al. (6)	TJR patients, NSQIP database, 2011–2017.	Demographics, comorbidities, functional status, and perioperative indicators (e.g., age, BMI, diabetes, ASA, modified frailty index).	Stratified sampling only
Gould et al. (7)	Primary TKA patients, single center, Australia, pre-March 2020.	Multidimensional variables encompassing biopsychosocial factors, comorbidities, prior healthcare utilization, and perioperative events.	Not explicitly mentioned
Klemt et al. (8)	Primary TKA, single center, 2016–2019.	Demographics, comorbidities, surgical specifics, and behavioral factors (e.g., insurance, anesthesia type, implant fixation, smoking/substance use).	Stratified sampling only
Wu et al. (9)	THA/hemiarthroplasty patients, single hospital, Taiwan, Sep 2016-Dec 2018.	Comorbidities, surgical factors, and laboratory values with a focus on coagulation profiles (e.g., CKD, CAD, INR, PT, aPTT) and history of falls.	SMOTE
Kunze et al. (10)	Medicare THA/TKA patients, claims data, Oct 2016–Sep 2018.	Encompassed patient demographics, hospital/surgeon procedural volumes, prior medical history, and county-level social determinants of health.	Downsampling
Shaikh et al. (11)	Elective THA/TKA patients, SPARCS database, 2012–2016.	Included demographics, socioeconomic factors, surgical context, and comorbidity burden quantified by the Elixhauser index.	Random Undersampling
Park et al. (12)	Primary THA/TKA, single academic safety-net hospital, 2016–2019.	Covered variables across the entire care continuum, from demographics and preoperative status to hospitalization and post-discharge factors, including patient-reported outcomes.	Not specified; oversampling; RUSBoost
Buddhiraju et al. (13)	Primary TKA patients, ACS-NSQIP database, 2013–2020.	Comprised core demographic and clinical variables: age, sex, BMI, comorbidities, preoperative labs, and hospitalization/surgical factors.	Not explicitly mentioned
Khan et al. (14)	Primary unilateral THA, US tertiary academic center, 2016–2020.	Featured a multidimensional set including demographics, Area Deprivation Index (ADI), comorbidities (CCI), patient-reported outcomes (PROMs), and perioperative details.	Not explicitly mentioned
Buddhiraju et al. (15)	Primary TKA patients, ACS-NSQIP, 2013–2020.	Consisted of preoperative demographic and clinical risk factors commonly used in surgical risk assessment (e.g., functional status, major organ system comorbidities).	Not explicitly mentioned
Khan et al. (16)	Primary unilateral TKA, US tertiary academic center, 2016-2020.	Included demographics, socioeconomic factors (e.g., ADI), comorbidities (CCI), patient-reported outcomes (PROMs), and perioperative details.	Not explicitly mentioned
Crespi et al. (17)	Primary THA patients, MARCQI, single institution, 2012–2023.	Comprised demographics, lifestyle factors, key comorbidities, and perioperative variables (e.g., LOS, discharge type, preoperative opioid use).	Not explicitly mentioned
Khan et al. (18)	Primary TKA patients, MARCQI center, 2016–2024.	Included demographics, lifestyle factors, key comorbidities, and perioperative variables (e.g., ASA grade, LOS, discharge disposition).	Not explicitly mentioned
Purbasari et al. (19)	THA patients, MIMIC-IV database.	Covered demographics, insurance type, a broad range of preoperative diagnoses, and implant-specific details (material and type).	Random oversampling (applied to training set)

BMI, body mass index; CHF, congestive heart failure; COPD, chronic obstructive pulmonary disease; CKD, chronic kidney disease; ASA, American Society of Anesthesiologist Physical Status score; CCI, Charlson comorbidity index; CAD, Coronary artery disease; PROM, Patient-reported outcome measure phenotype; LOS, Length of stay; NARX, Opioid overdose risk score; DVT/PE, deep vein thrombosis or pulmonary embolism.

**TABLE 2:** Study data.

Study	Type	Length (Day)	N	N. events	Models	Auc
Mohammadi et al. (5)	THA	30	478	∼55	ANN	0.846(0.823–0.871)
	TKA		628	∼73	ANN	0.822(0.809–0.862)
Slezak et al. (6)	TKA/THA	30	∼95523	∼3486	RF	0.602(0.593–0.613)
					LR	0.658(0.648–0.668)
					XGBoost	0.673(0.663–0.683)
					LightGBM	0.672(0.662–0.682)
Gould et al. (7)	TKA	30	∼921	∼63	RF	0.692(0.621–0.764)
					LR	0.589(0.506–0.673)
Klemt et al. (8)	TKA	90	2005	∼129	ANN	0.85(0.81–0.89)
					SVM	0.82(0.78–0.86)
					KNN	0.82(0.78–0.83)
					LR	0.83(0.79–0.87)
Wu et al. (9)	THA	30	296	8	LR	0.982
					DT	0.933
					RF	0.976
					ANN	0.989
Kunze et al. (10)	TKA	30	87,930	3,517	GAM	0.66
	THA		47,878	1,915	GAM	0.65
Shaikh et al. (11)	TKA/THA	30	6000	∼176	AB	0.48
					XGBoost	0.62(0.62–0.63)
					LR	0.61(0.61–0.62)
					RF	0.68(0.67–0.68)
					SVM	0.63(0.63–0.64)
					1 Layer NN	0.53(0.53–0.54)
					5 Layer NN	0.53(0.53–0.54)
		90	6000	∼25	AB	0.72(0.72–0.73)
					XGBoost	0.61(0.6–0.62)
					LR	0.62(0.62–0.63)
					RF	0.69(0.68–0.69)
					SVM	0.64(0.63–0.65)
					1 Layer NN	0.53(0.53–0.54)
					5 Layer NN	0.53(0.53–0.54)
Park et al. (12)	TKA/THA	90	∼397	∼23	LR	0.845(0.825–0.87)
					LASSO	0.862(0.842–0.885)
					SVM-polynomial	0.714(0.705–0.789)
					SVM-radial	0.789 (0. 771–0.839)
					RF	0.836(0.819–0.855)
					RF w/oversampling	0.835(0.816–0.85)
					RUS boosting	0.835(0.811–0.863)
Buddhiraju et al. (13)	TKA	30	73157	2,297	ANN	0.78
					RF	0.78
					HGB	0.78
					NEPLR	0.78
Khan et al. (14)	THA	30	8893	310	LR	0.7(0.67–0.72)
		90		502	LR	0.71(0.69–0.74)
Buddhiraju et al. (15)	TKA	30	2835	88	ANN	0.72
Khan et al. (16)	TKA	30	10521	444	LR	0.65(0.57–0.75)
		90		704	LR	0.68(0.67–0.7)
Crespi et al. (17)	THA	90	402	21	MPNN	0.71
Khan et al. (18)	TKA	90	645	26	MPNN	0.704
Purbasari et al. (19)	THA	365	346	71	XGB	0.996
					SGB	0.994
					RF	0.994
					SVM	0.991
					DT	0.878
					MLP	0.95
					LR	0.662

RF, random forest; LR, logistic regression; SVM, support vector machine; NN, neural network; KNN, k-nearest neighbors; DT, decision tree; GB, gradient boosting.

The studies were published between 2020 and 2025. Study populations primarily comprised patients undergoing primary THA and/or TKA. Data sources were heterogeneous, including single-center Electronic Health Records (EHRs), multi-center registries [e.g., the American College of Surgeons National Surgical Quality Improvement Program (ACS-NSQIP), Statewide Planning and Research Cooperative System (SPARCS)], and administrative claims databases (e.g., Medicare) (5, 6, 10, 11).

A wide array of ML algorithms was employed. Commonly used models included traditional methods like Logistic Regression (LR) (6–9, 11–14, 16, 19) and ensemble methods like Random Forest (RF) (6, 7, 9, 11–13, 19), as well as more complex algorithms such as Artificial Neural Networks (ANNs) (5, 8, 9, 13, 15), Support Vector Machines (SVMs) (8, 11, 12, 19), gradient boosting machines (e.g., XGBoost (6, 11, 19), LightGBM) (6), and novel architectures like Message Passing Neural Networks (MPNNs) (17, 18). Model performance, evaluated primarily by the Area Under the Receiver Operating Characteristic Curve (AUC), varied substantially, with reported values ranging from approximately 0.48 (11) to over 0.99 (19).

Various strategies were employed to address class imbalance, including undersampling (5, 11), oversampling (12, 19), the Synthetic Minority Over-sampling Technique (SMOTE) (9), and ensemble methods like RUSBoost (13). Several studies did not specify their approach to handling imbalance (7, 13–18).

Predictors spanned multiple domains: demographic factors, comorbidities (using indices like the Charlson Comorbidity Index or specific diagnoses), surgical details, laboratory values, and socioeconomic indicators (5–19). The outcome was unplanned readmission within a defined postoperative window, most commonly 30 days (5–7, 9–11, 13–16), followed by 90 days (8, 11, 12, 14, 16–18); one study used a 365-day window (19). Sample sizes varied from 296 (9) to over 95,000 (6), with corresponding readmission event counts from single digits (9) to several thousand (6, 10).

### 3.3 Meta-analysis, heterogeneity, and publication bias

**FIGURE 2:**
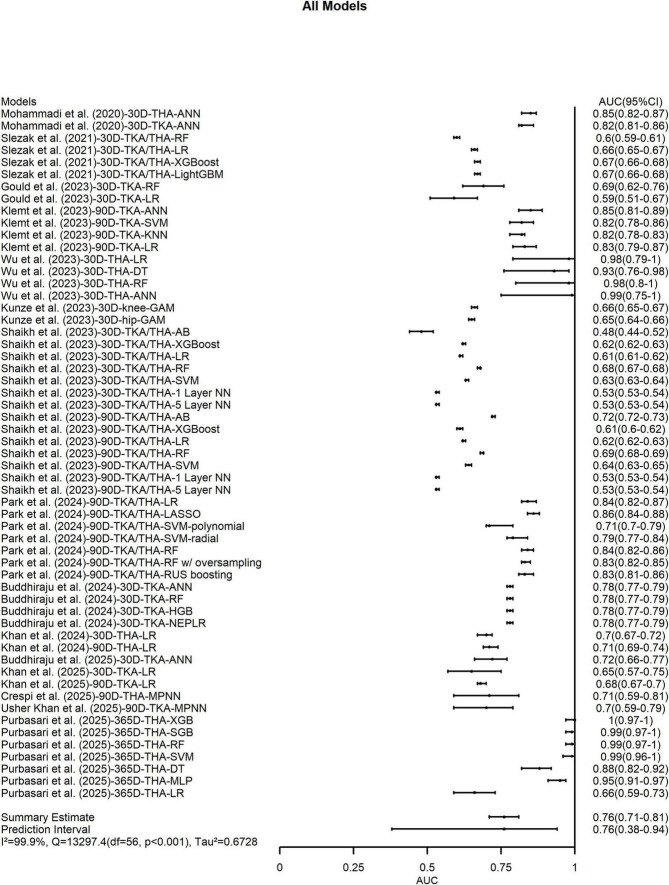
Meta-analysis.

The meta-analysis yielded a pooled summary estimate (logit AUC) of 0.76 (95% CI: 0.71–0.81) (Figure 2). However, given the extreme between-model heterogeneity (I^2^ = 99.9%, Tau^2^ = 0.6728), this summary estimate is of limited clinical interpretability as it represents a mathematical average rather than a reproducible performance benchmark. More germane to clinical translation, the 95% prediction interval (0.38–0.94) indicates that the true performance of a model developed in a new setting is highly uncertain, potentially ranging from poor to excellent. This wide interval underscores the profound impact of contextual and methodological moderators on model generalizability.

**FIGURE 3:**
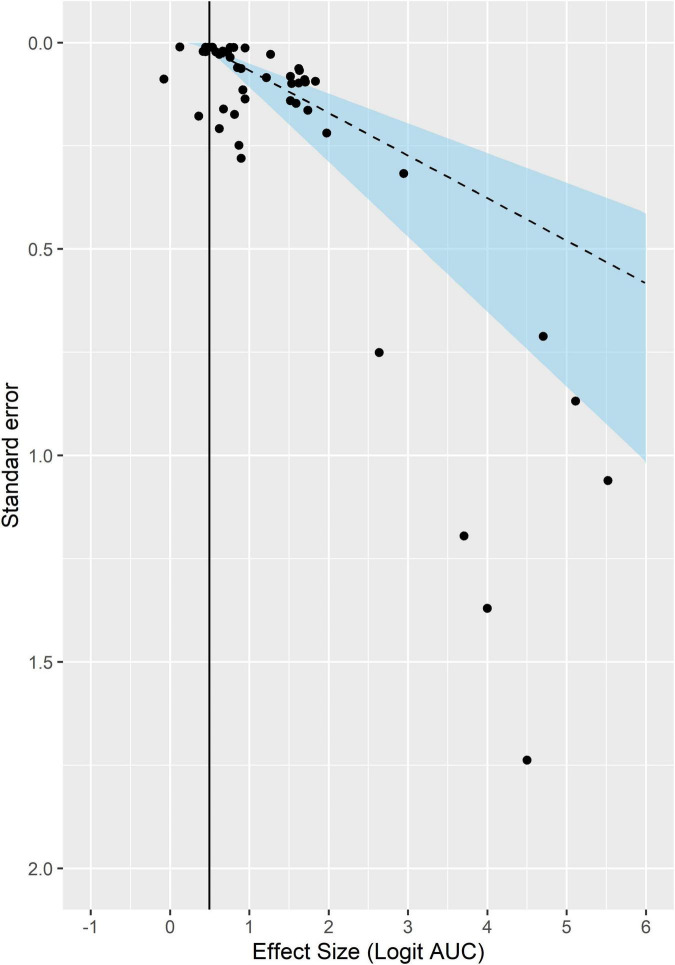
Funnel plot.

Visual inspection of the funnel plot (Figure 3) showed marked asymmetry. High-precision studies clustered near the summary estimate, while lower-precision studies exhibited a wider, more scattered distribution of effect sizes, with several falling below the pooled estimate. This pattern, alongside the extreme statistical heterogeneity, indicates substantial genuine differences between studies beyond sampling error, likely due to unaccounted moderating factors explored in subgroup analyses. While publication bias cannot be ruled out, the observed asymmetry is more strongly attributable to this high heterogeneity.

### 3.4 Subgroup analyses

**FIGURE 4:**
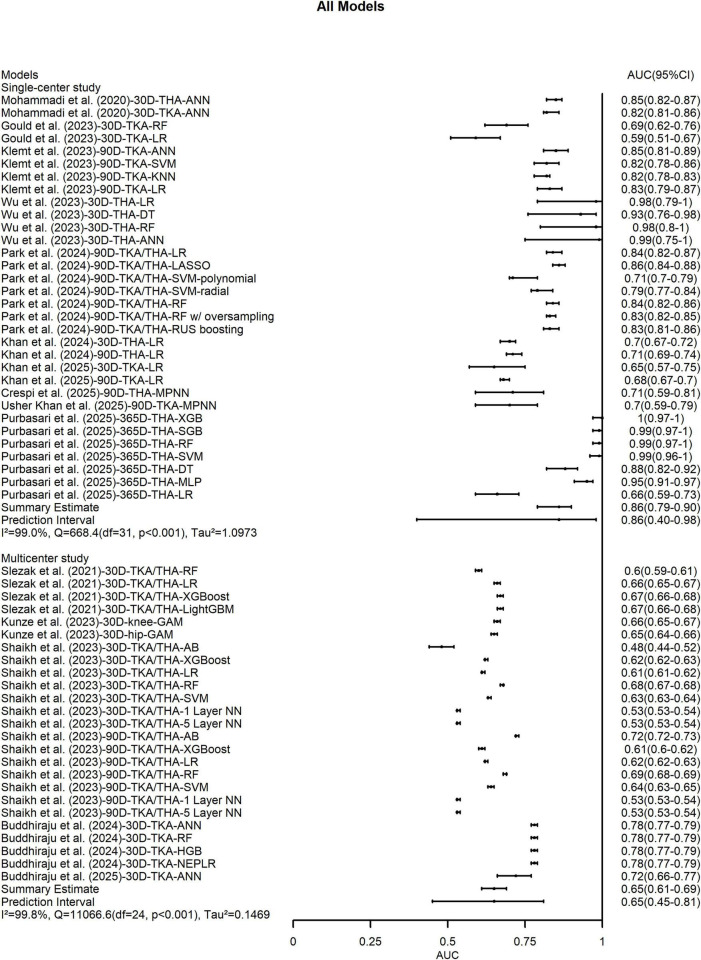
Subgroup analyses: study design.

Study Design (Single-Center vs. Multicenter): Models from single-center studies (10 studies; *k* = 32) showed a pooled estimate of 0.86 (95% CI: 0.79–0.90), while those from multicenter studies (4 studies; *k* = 25) showed a significantly lower estimate of 0.65 (95% CI: 0.61–0.69) (Figure 4). Heterogeneity remained extreme within both subgroups (*I*^2^ = 99.0 and 99.8%, respectively).

**FIGURE 5:**
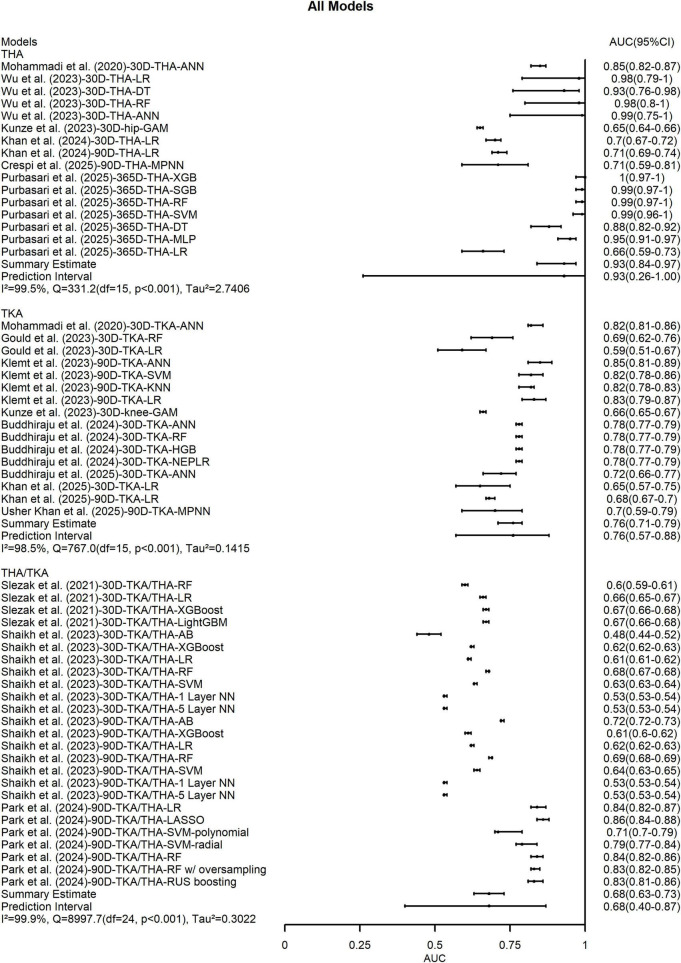
Subgroup analyses: surgical procedure.

Surgical procedure: THA-specific models (from 6 independent studies; *k* = 16) yielded a pooled estimate of 0.93 (95% CI: 0.84–0.97). TKA-specific models (7 studies; *k* = 16) showed 0.76 (95% CI: 0.71–0.79), while mixed-cohort models (3 studies; *k* = 25) yielded 0.68 (95% CI: 0.63–0.73) (Figure 5). Heterogeneity remained extreme (*I*^2^ > 98%).

**FIGURE 6:**
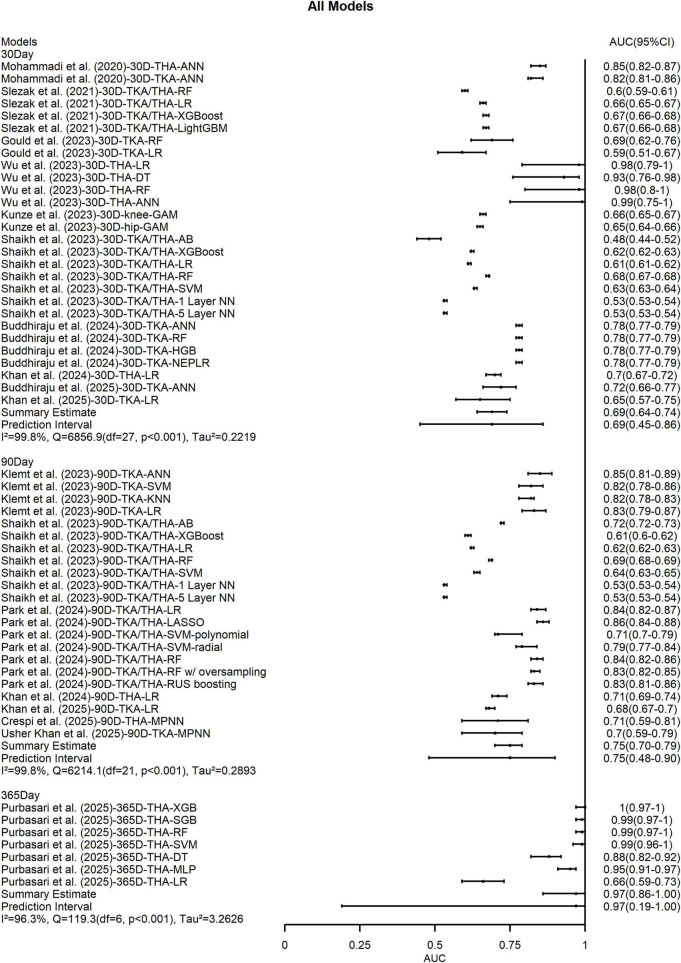
Subgroup analyses: prediction timeframe.

Prediction timeframe: Models for 30-day prediction (10 studies; *k* = 28) yielded a pooled estimate of 0.69 (95% CI: 0.64–0.74). Performance appeared higher for 90-day models (7 studies; *k* = 22; 0.75, 95% CI: 0.70–0.79) and 365-day models (1 study; *k* = 7; 0.97, 95% CI: 0.86–1.00) (Figure 6) (Exploratory; based on a single study). Extreme heterogeneity persisted across all timeframes (I^2^ from 96.3 to 99.8%). Crucially, given the reliance on a single study, the 365-day estimate represents a hypothesis-generating observation rather than a stable performance benchmark and should be interpreted with extreme caution.

**FIGURE 7:**
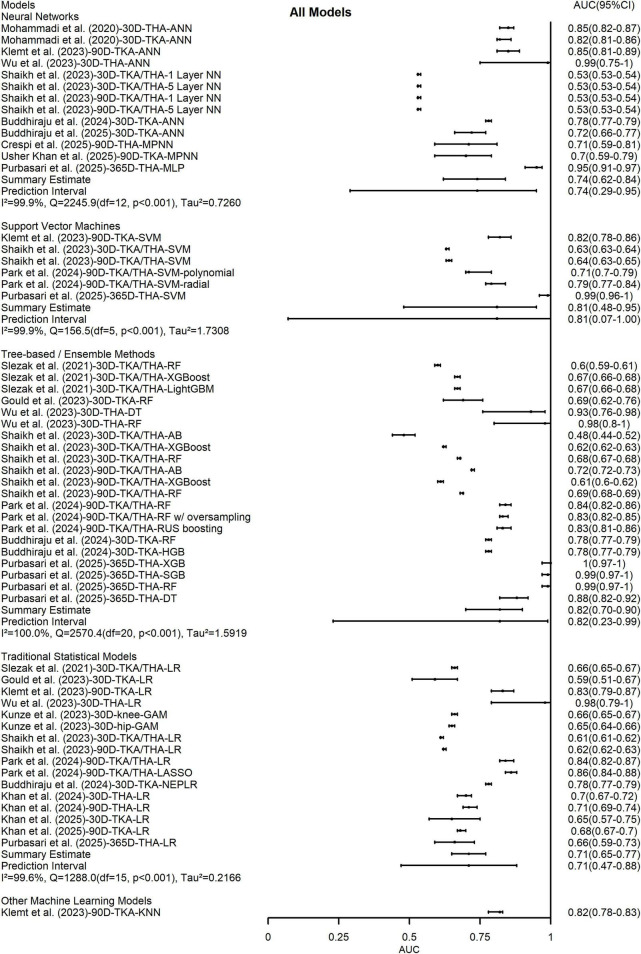
Subgroup analyses: model class.

Model class (algorithm type): Support Vector Machines (4 studies; *k* = 6) and Tree-based/Ensemble Methods (7 studies; *k* = 21) had the highest pooled point estimates (0.81 and 0.82, respectively), while Traditional Statistical Models (11 studies; *k* = 16) had a lower estimate (0.71, 95% CI: 0.65–0.77) (Figure 7). Neural Networks (9 studies; *k* = 13) showed an estimate of 0.74 (95% CI: 0.62–0.84). Heterogeneity was extreme across all algorithm subgroups (I^2^ from 99.6 to 100.0%).

**FIGURE 8:**
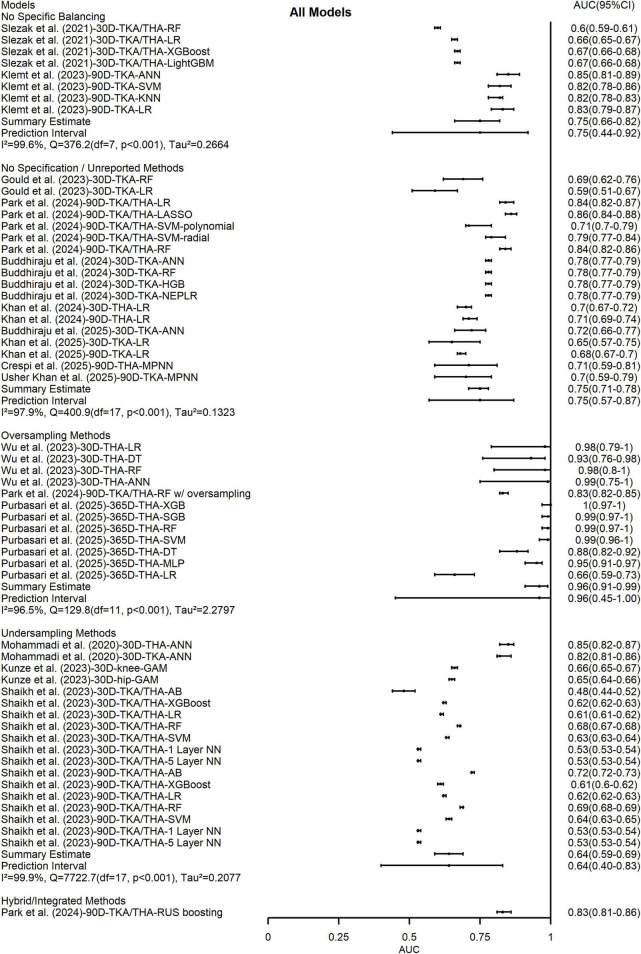
Subgroup analyses: class imbalance handling method.

Class imbalance handling method: Models from studies using oversampling (3 studies; *k* = 12) had the highest pooled estimate (OR: 0.96, 95% CI: 0.91–0.99), while those using undersampling (3 studies; *k* = 18) had the lowest (OR: 0.64, 95% CI: 0.59–0.69) (Figure 8). Models with no balancing (2 studies; *k* = 8) or unreported methods (8 studies; *k* = 18) showed intermediate performance (OR: 0.75 for both). High heterogeneity persisted within each subgroup.

### 3.5 Risk of bias and applicability

**FIGURE 9:**
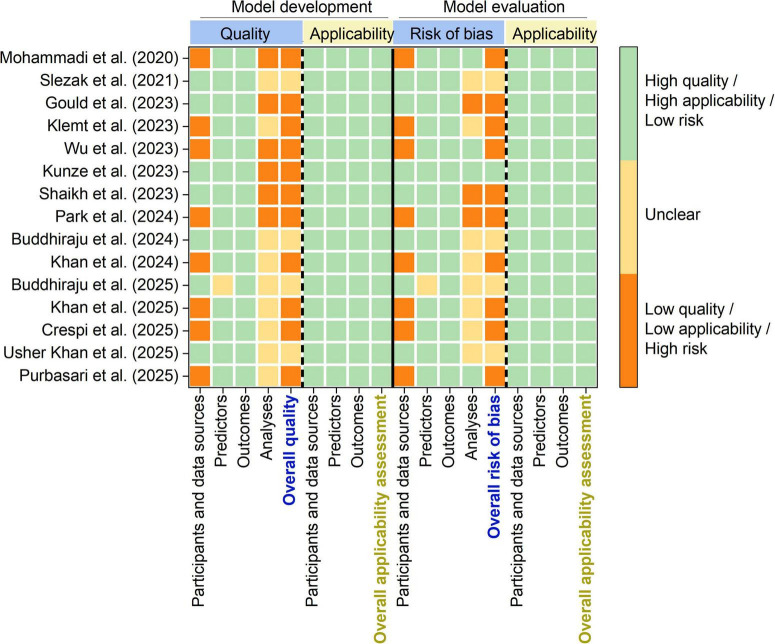
Risk of bias and applicability.

Assessment using the PROBAST + AI tool (Figure 9) indicated a high overall risk of bias for most studies, primarily in the Analysis domain and the Participants domain during model development. Common concerns included lack of sample size justification, inadequate reporting on handling missing data, absence of model recalibration when class imbalance techniques were used, and incomplete description of resampling procedures. The risk of bias was generally low for the Predictors and Outcome domains. Concerns regarding the applicability of the studies to the review question were low across all domains.

## 4 Discussion

This systematic review and meta-analysis synthesizes evidence from 15 studies investigating ML-based prediction models for readmission after THA/TKA. While the pooled C-statistic was 0.76 (95% CI: 0.71–0.81), the extreme statistical heterogeneity (*I*^2^ = 99.9%) renders this point estimate of limited clinical utility. The clinically actionable insight is encapsulated by the 95% prediction interval (0.38–0.94), which highlights the profound unpredictability of model performance. Consequently, our subgroup analyses were not undertaken to derive precise performance benchmarks—which would be implausible given the limited number of independent studies (e.g., *n* = 1 for 365-day models, *n* = 6 for THA-specific models)—but rather to identify potential methodological and clinical moderators that drive this wide variation.

Our subgroup analyses provide crucial insights into the sources of this variability. First, study design emerged as a strong moderator. Despite the limited number of multicenter studies (*n* = 4), models derived from single-center data reported significantly higher—and likely optimistic—performance estimates (pooled AUC 0.86) compared to those developed from multicenter data (pooled AUC 0.65). This discrepancy aligns with the known challenge of overfitting to local data patterns and underscores the greater potential for generalization, albeit with more conservative performance metrics, of models trained on diverse, multi-institutional cohorts (11, 13). Second, the target surgical procedure significantly influenced the results. THA-specific models (6 studies) demonstrated notably higher discriminative ability than TKA-specific models (7 studies) or those built on mixed cohorts (3 studies). However, given the small number of contributing studies and overlapping confidence intervals, this difference should be interpreted as hypothesis-generating rather than definitive evidence of superior THA model performance. This may reflect inherent differences in the pathophysiology leading to readmission, variability in population homogeneity, or differences in the predictive utility of available variables for each procedure (17, 28), supporting the argument for procedure-specific model development. Third, while a trend toward higher AUCs was observed with longer prediction timeframes, this finding is severely limited by the fact that the 365-day estimate derives entirely from a single study. Although this seemingly counterintuitive trend may hypothetically reflect that longer-term readmissions are more strongly driven by stable patient factors (e.g., chronic comorbidities), this observation currently lacks the empirical robustness to support definitive conclusions regarding temporal model performance. In contrast, short-term readmissions may be more influenced by acute, peri-operative, or surgical factors that are harder to predict consistently (29). Emerging evidence highlights neurocognitive complications—including postoperative delirium and perioperative neurocognitive disorders—as prominent acute peri-operative drivers of early readmission after arthroplasty (30). Randomized trial protocols indicate that interventions such as repetitive transcranial magnetic stimulation to mitigate postoperative delirium (31) and opioid-free anesthetic regimens to reduce neurocognitive deficits in elderly hip fracture surgery (32) may alter readmission risk profiles. Incorporating neurocognitive risk factors or intervention exposure as candidate predictors in future ML models could therefore improve the capture of acute peri-operative contributors to short-term readmission, addressing a key gap in current model performance.

Regarding modeling techniques, this study observed that advanced ML algorithms (e.g., SVM, tree-based ensembles) did not demonstrate a consistent or decisive superiority over traditional statistical models like logistic regression. In fact, logistic regression models showed more consistent performance (lower Tau^2^) across studies, albeit at a modest average level. This finding echoes a growing consensus in predictive analytics that increased model complexity does not automatically translate to superior clinical utility, particularly with limited sample sizes or high-dimensional, noisy data (33, 34). Furthermore, the method for handling class imbalance was identified as a critical and frequently under-reported methodological factor. The use of oversampling techniques was associated with optimistically high performance estimates (9, 19). A concomitant and concerning finding was the near-universal lack of model recalibration following the application of such techniques. This oversight can lead to poorly calibrated probability estimates—where predicted risks do not match observed event rates—severely undermining the models’ practical utility for individualized clinical risk stratification (35).

The PROBAST + AI assessment confirmed a high risk of bias in the majority of included studies, with concerns concentrated primarily in the Analysis domain. Common shortcomings—including inadequate handling of missing data, lack of sample size justification, and poor reporting of resampling—represent critical threats to internal validity. Crucially, these pervasive methodological weaknesses are not merely background noise; they are a primary driver of the extreme heterogeneity (*I*^2^ = 99.9%) observed in our synthesis. While the predictors and outcomes were largely applicable, the prevailing biases significantly curb confidence in the reported performance estimates. Consequently, the current evidence base is too unreliable to support the clinical translation of these specific models, regardless of their theoretical potential.

Several limitations of this review should be acknowledged. First, while 15 included studies and 57 distinct ML models provided sufficient data points to explore sources of heterogeneity, the small number of independent publications limited the statistical power for definitive subgroup comparisons. Formal interaction tests were not feasible due to extreme heterogeneity. Crucially, several key subgroups were represented by very few studies (e.g., only 1 study contributed to the 365-day models, and only 3–6 studies defined procedure-specific cohorts). Therefore, all subgroup findings—including the apparently high AUC for THA or 365-day models—must be interpreted strictly as exploratory and hypothesis-generating. The observed trends offer insights into potential moderators but do not constitute conclusive evidence of performance differences. Nevertheless, the observed trends—such as the significant performance gap between single-center and multicenter models—offer critical insights into model generalizability. Second, the persistently high heterogeneity, even within our defined subgroups, suggests the influence of important unmeasured or unexamined moderators. These could include specific data quality and curation practices, granular details of feature engineering, or hyperparameter optimization strategies. Third, the observed asymmetry in the funnel plot raises the possibility of publication bias, where studies reporting lower model performance may be under-represented in the published literature. This could lead to an overestimation of the true average discriminative ability in the field. Fourth, our quantitative synthesis focused solely on the C-statistic (discrimination) because a meaningful pooled analysis of calibration metrics was precluded by inconsistent and incomplete reporting across studies. This represents a critical evidence gap in the field of orthopedic ML research. While discrimination (AUC) quantifies a model’s ability to rank patients, it fails to assess whether predicted risks match observed event rates. Without transparent reporting of calibration plots, intercept, slope, and Brier scores, even a model with high AUC may produce systematically biased probability estimates (e.g., predicting a 5% risk when the true risk is 20%), thereby undermining safe clinical decision-making. We strongly advocate that future studies adhere to the TRIPOD-AI reporting guideline to ensure both discrimination and calibration are rigorously evaluated and transparently reported.

## 5 Conclusion

In conclusion, while machine learning models for THA/TKA readmission suggest preliminary promise, this potential is currently severely constrained by prevalent methodological limitations and a high risk of bias. The pooled analysis indicates a moderate average discriminative ability; however, the performance of any individual model is highly contingent on specific contextual and methodological factors, including study design (single-center vs. multicenter), target surgical procedure, prediction timeframe, and approaches to handling class imbalance. There is no consistent evidence that complex ML algorithms confer a definitive advantage over traditional statistical models like logistic regression in this context. To advance the field, future research should prioritize the development and validation of models using robust, multi-institutional datasets, adhere to stringent methodological and reporting standards (e.g., TRIPOD-AI), ensuring the transparent and mandatory reporting of both discrimination and calibration metrics (e.g., calibration slopes, intercepts, and Brier scores), and proactively address class imbalance with subsequent model recalibration. Closing this calibration reporting gap is paramount before these tools can be safely translated to bedside risk stratification. Before any consideration for widespread clinical implementation, promising prediction models require rigorous external validation in diverse, real-world settings to demonstrate robust, generalizable, and clinically useful performance.

## Data Availability

The original contributions presented in this study are included in the article/Supplementary material, further inquiries can be directed to the corresponding author.
