# Supplementary material for: Systematic review and meta-analysis of machine learning-based prediction models for readmission risk after total hip and knee arthroplasty
Source: Front Med (Lausanne). 2026 Jul 15;13:1855638. doi: 10.3389/fmed.2026.1855638 (PMC13415355; doi:10.3389/fmed.2026.1855638)
Supplement: Supplementary file 1 [file Table_1.docx]

**Supplementary Table 1 Consistency Test for Quality Evaluation Results**

| **Model Development** | | | | | | | |
| --- | --- | --- | --- | --- | --- | --- | --- |
|  | **Quality** | | | | **Applicability** | | |
|  | **Participants** | **Predictors** | **Outcomes** | **Analyses** | **Participants** | **Predictors** | **Outcomes** |
| **Kappa value** | 0.602 | 0.643 | 1 | 0.868 | 1 | 1 | 1 |
| ***P*** | 0.0187 | <0.001 | <0.001 | <0.001 | <0.001 | <0.001 | <0.001 |
| **Model Evaluation** | | | | | | | |
|  | **Risk of bias** | | | | **Applicability** | | |
|  | **Participants** | **Predictors** | **Outcomes** | **Analyses** | **Participants** | **Predictors** | **Outcomes** |
| **Kappa value** | 0.737 | 0.464 | 1 | 0.779 | 1 | 1 | 1 |
| ***P*** | 0.0031 | 0.0018 | <0.001 | <0.001 | <0.001 | <0.001 | <0.001 |

**Note:** *P* < 0.05 indicates that the results of the two evaluators are consistent.
